# Patient experience with NER1006 as a bowel preparation for colonoscopy: a prospective, multicenter US survey

**DOI:** 10.1186/s12876-021-01605-y

**Published:** 2021-02-15

**Authors:** Brooks D. Cash, Mary Beth C. Moncrief, Michael S. Epstein, David M. Poppers

**Affiliations:** 1 University of Texas Health Science Center, Houston, USA; 2 Synchrony Medical Communications, LLC, West Chester, PA USA; 3 Investigative Clinical Research and Digestive Disorders Associates, Annapolis, MD USA; 4 New York University Langone Health, New York, NY USA

**Keywords:** Bowel preparation, Cathartics, NER1006, Patient satisfaction, Plenvu, Survey, Treatment adherence

## Abstract

**Background:**

NER1006 (Plenvu^®^, Salix Pharmaceuticals, Bridgewater, NJ) is a 1 L polyethylene glycol bowel preparation indicated for colonoscopy in adults. A US online survey assessed real-world ease of use and treatment satisfaction in individuals who received NER1006.

**Methods:**

Adults were recruited from 444 US community gastrointestinal practices and provided a kit number for enrollment into an online survey to be completed within 2 weeks. Survey questions evaluated colonoscopy history and prior bowel preparation(s) prescribed, patient experience during NER1006 administration, and patient satisfaction with the bowel preparation process. A 9-point predefined grading scale was used to evaluate ease of NER1006 preparation and consumption (range, 1 “very difficult” to 9 “very easy”); the perceived importance of volume requirement and clear liquid options (range, 1 “not important at all” to 9 “very important”); and patient satisfaction (range, 1 “not satisfied at all” to 9 “very satisfied”).

**Results:**

1630 patients were enrolled, 1606 underwent colonoscopy, and 1598 completed the survey between September 15, 2018 and February 28, 2019. Among 1606 patients who had a colonoscopy, 62.5% were female, and the mean patient age was 54.4 years (range 18–89 years). Most patients (74.7%) did not report a family history of colon cancer, 62.6% had undergone prior colonoscopy, and 64.8% were undergoing colonoscopy for routine colorectal cancer screening. A majority (76.1%) of patients who completed the survey reported that NER1006 was very easy to prepare and take, and 89.9% were very or moderately satisfied with NER1006 overall. Most (97.6%) patients reported consuming all or most of the bowel preparation. Among 1005 patients with previous bowel preparation use, 84.7% indicated that their experience with NER1006 was much better or better (65.3%) or about the same (19.4%) compared with previously used bowel preparations, while only 15.3% rated NER1006 as worse or much worse.

**Conclusions:**

In this first real-world, US multicenter survey, patient-reported experience with NER1006 as a bowel preparation for colonoscopy was favorable and adherence was high. The majority of patients were very or moderately satisfied with the overall experience and found it much better/better than previously used bowel preparations.

*Trial registration*: Not applicable

## Background

Colonoscopy is the most common endoscopic procedure in the United States, with approximately 11 million performed for adults in 2013 [1]. Of 1.5 million patients aged 50–75 years at average risk of developing colorectal cancer (CRC) who underwent screening colonoscopy as part of the Gastrointestinal Quality Improvement Consortium, 34.6% underwent removal of ≥ 1 adenomatous polyp [1]. A 54% reduction in US CRC-related mortality rates has been observed from 1970 to 2017, and screening for CRC has been a key factor in this trend [2]. However, findings from the 2015 National Health Interview Survey showed that nearly 40% of eligible individuals were nonadherent to CRC screening guidelines [3]. In one single-center study (*N* = 617), the odds of nonadherence to a scheduled colonoscopy were significantly greater for a screening colonoscopy compared with a surveillance colonoscopy; that is, a colonoscopy performed with a known patient medical history of adenomas (odds ratio [OR], 12.7; 95% confidence interval [CI] 4.2–38.5) [4]. Results from a single-center prospective study indicated that bowel preparation was cited as the most common deterrent to undergoing a screening colonoscopy for individuals who had never undergone CRC screening (66% of 126 patients), as well as those previously screened (57% of 132 patients) [5].

Adequate bowel preparation is critical for colonoscopy success [6]. A number of factors have been associated with inadequate bowel preparation, including failure to follow preparation instructions (OR, 2.7; 95% CI 1.5–4.8; *P* = 0.001) and male sex (OR, 1.5; 95% CI 1.03–2.3; *P* = 0.04) [6]. Bowel preparation–related factors that have been shown to impact preparation quality include dosing regimen (e.g., split-dosing, timing between preparation completion and colonoscopy), the volume of the bowel preparation required to be ingested by the patients, and preparation palatability [7–9].

The first low-volume 1 L polyethylene glycol (PEG)–based bowel preparation, NER1006 (Plenvu^®^, Salix Pharmaceuticals, Bridgewater, NJ), was approved in the United States in 2018 and is indicated for colon cleansing in preparation for colonoscopy in adults [10]. In the United States, NER1006 may be administered as a 2-day split-dose (i.e., evening before/morning of [pm/am] the colonoscopy) or as a 1-day split-dose regimen on the morning of the colonoscopy (am/am) [10]. The 2 doses of NER1006 consist of mango (dose 1) and fruit punch (dose 2) flavors [10] to minimize the risk of “taste fatigue.” The efficacy, safety, and tolerability of NER1006 has been demonstrated in three phase 3, randomized, active comparator, noninferiority trials [11–13]. Patient diary data from the trials supported the ease of following the NER1006 bowel preparation instructions, ease of consumption, and acceptability of bowel preparation taste [11–13]. Because data on patient experiences with NER1006 in a community clinical practice setting are lacking, the current prospective US survey was conducted to assess real-world patient experience with respect to ease of use and treatment satisfaction with NER1006 in adults.

## Methods

Men and women ≥ 18 years of age scheduled for a colonoscopy at community gastroenterology practices in the United States were recruited to participate in a patient survey and were provided with a kit number to enroll online. Patients received a sample of the bowel preparation NER1006 and were given instructions from their health care providers on dosing and administration according to the US prescribing information [10], which allows for a 2-day pm/am split-dose or 1-day am/am split-dose administration. For the pm/am dosing regimen, on the evening (~ 4:00 pm to 8:00 pm) before the colonoscopy, dose 1 of NER1006 was mixed with ≥ 16 oz of water and consumed during a 30-min time period, followed by ≥ 16 oz of clear liquid consumed during a 30-min period, with consumption of additional clear liquids recommended during the evening. Approximately 12 h after the start of dose 1, dose 2 of NER1006 was mixed with ≥ 16 oz of water and consumed during a 30-min period, followed by ≥ 16 oz of clear liquid consumed during a 30-min period. For the am/am dosing regimen, dose 1 of NER1006 was mixed with ≥ 16 oz of water and consumed during a 30-min time period, followed by ≥ 16 oz of water consumed during a 30-min period. Starting ≥ 2 h after the start of dose 1, dose 2 of NER1006 was mixed with ≥ 16 oz of clear liquid and consumed during a 30-min period, followed by ≥ 16 oz of clear liquid consumed during a 30-min period. For both NER1006 dosing regimens, patients were advised not to consume additional oral intake (liquids or solids) within 2 h before the colonoscopy.

Within 2 weeks after the colonoscopy, an online survey specifically designed for this study was to be completed by the patients (survey questions are provided in Additional file 1: Appendix). Each patient who completed the survey received modest monetary compensation for their time. Online survey questions focused on an individual’s history of colonoscopy and prior bowel preparation(s) prescribed, experience during administration of NER1006, and patient satisfaction with the bowel preparation process. Nine-point predefined grading scales were used to evaluate the ease of NER1006 preparation and consumption (range, 1 “very difficult” to 9 “very easy”), the importance of volume requirement and importance of clear liquid choice (range, 1 “not important at all” to 9 “very important”), and patient satisfaction (range, 1 “not satisfied at all” to 9 “very satisfied”). For importance and satisfaction survey questions, scores were grouped into 3 classifications: 7–9 very satisfied or important, 4–6 moderately satisfied or important, and 1–3 not satisfied or important. Data were deidentified prior to analyses and summarized using descriptive statistics.

## Results

A total of 707 health care providers from 444 community practices participated, and 1630 patients were enrolled in the survey. Of these 1630 patients, 1606 (98.5%) underwent colonoscopy, and 1598 (98.0%) completed the survey between September 15, 2018 and February 28, 2019. Of the 1606 patients who underwent colonoscopy, the majority (62.5%) were female, and the mean age was 54.4 years (range 18–89 years; Table 1). While data were unknown for 8 of the 1606 patients, most (74.7%) reported that they did not have a family history of CRC, 62.6% had previously used a bowel preparation for colonoscopy, and 64.8% were undergoing colonoscopy for routine CRC screening. Most (91.6%) of the 1598 patients who completed the survey received NER1006 as a pm/am dosing regimen, and most (97.6%) patients reported consuming all or most of the bowel preparation (Table 2) [14].

**Table 1:** Demographics and baseline characteristics

Parameter	Patients, n (%)
	*n* = 1606
Age, y	
Mean (SD)	54.4 (13.9)
Range	18–89
Sex	
Male	561 (34.9)
Female	1003 (62.5)
Not reported	42 (2.6)
Geographic area	
Northeast	390 (24.3)
Midwest	227 (14.1)
South	748 (46.6)
West	241 (15.0)
Family history of colon cancer	
Yes	399 (24.8)
No	1199 (74.7)
Not reported	8 (0.5)
First (initial/baseline) colonoscopy	
Yes	593 (36.9)
No	1005 (62.6)
Not reported	8 (0.5)
Indication for colonoscopy	
Diagnostic	557 (34.7)
Routine screening	1041 (64.8)
Not reported	8 (0.5)

**Table 2:** Patient survey results

Topic and response categories	Patients, n (%)
	*n* = 1598
Dosing of NER1006	
2-day pm/am dosing regimen	1463 (91.6)
1-day am/am dosing regimen (day of colonoscopy)	135 (8.4)
Volume of bowel preparation regimen completed	
All	1392 (87.1)
Most	167 (10.5)
At least half	29 (1.8)
Less than half	10 (0.6)
“How easy was it for you to prepare and take [NER1006]?”	
Very easy (score, 7–9)	1216 (76.1)
Medium level of difficulty (score, 4–6)	245 (15.3)
Very difficult (score, 1–3)	137 (8.6)
“How important is it to you that [NER1006] can be taken with your choice of clear liquids?”	
Very important (score, 7–9)	1192 (74.6)
Medium importance (score, 4–6)	273 (17.1)
Not important (score, 1–3)	133 (8.3)
“Would you be willing to recommend [NER1006] to family/friends?”	
Yes	1027 (64.3)
Maybe	414 (25.9)
No	157 (9.8)
“How was your experience with [NER1006] compared to the other bowel cleansing medications(s) you previously used?^a^	*n* = 1005
Much better/better	656 (65.3)
About the same	195 (19.4)
Worse/much worse	154 (15.3)

^a^For the 1005 patients for whom this was not their first colonoscopy, prior bowel cleansing medications were: 4 L PEG (23.5%; any formulation), oral sodium sulfate (17.1%), over-the-counter agent(s) (12.8%), 2 L PEG (11.4%), other (13.0%), or unknown (39.4%). Patients may have selected > 1 prior bowel preparation. PEG: polyethylene glycol. Used with permission from Cash B. and Moncrief MBC, abstract 534. Am J Gastroenterol. 2019; 114:S36 [14]

**Fig. 1:**
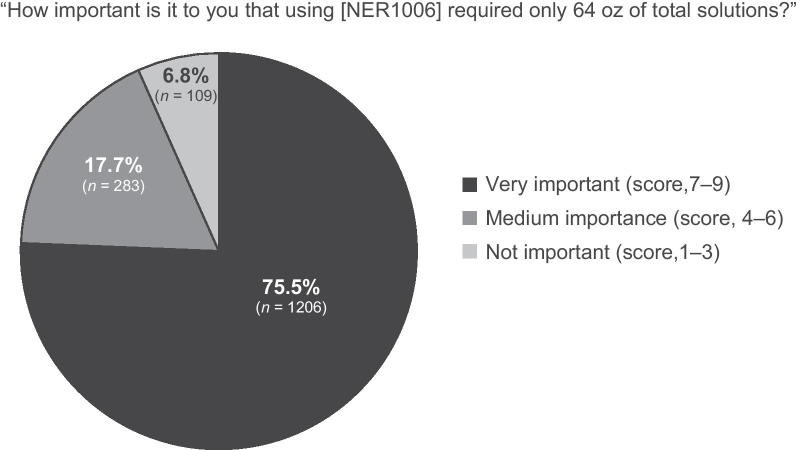
Patient response on importance of total volume requirement. *N* = 1598

**Fig. 2:**
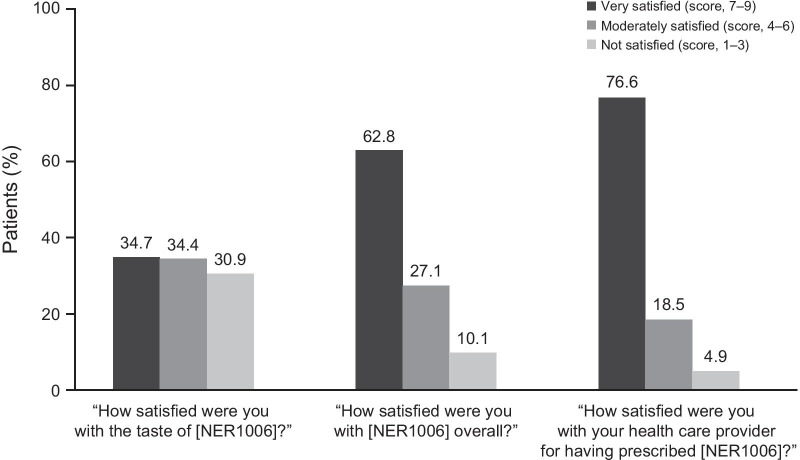
Patient satisfaction. *N* = 1598; used with permission from Cash B. and Moncrief MBC, abstract 534. Am J Gastroenterol. 2019; 114:S36 [14]

A majority (76.1%) of patients reported that NER1006 was very easy to prepare and take (Table 2), and most (75.5%) stated it was very important that NER1006 only required 64 oz of total solution volume (Fig. 1). In addition, 89.9% were very or moderately satisfied with NER1006 overall, and 95.1% were very satisfied or moderately satisfied with their health care provider for having prescribed NER1006 (Fig. 2) [14].

**Fig. 3:**
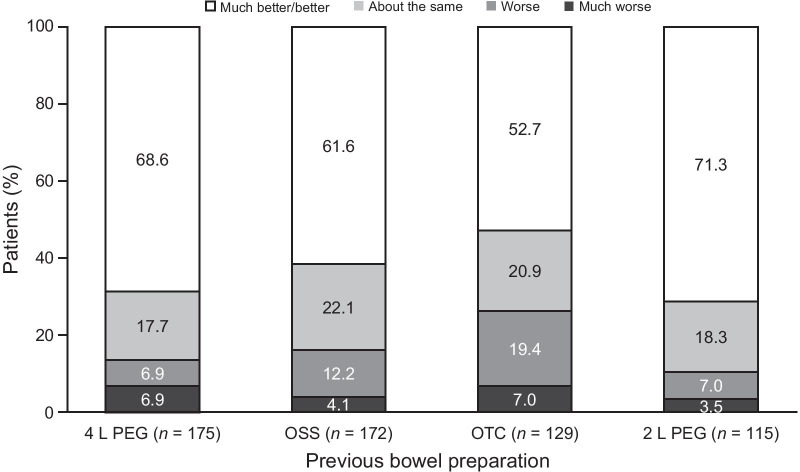
NER1006 experience compared with other bowel cleansing medications previously used. Patients may have selected > 1 prior bowel preparation, 4 L PEG data are for GoLYTELY^®^ (Braintree Laboratories, Inc., Braintree, MA, United States), and not all data are shown (other bowel preparation used or data not available). OSS: oral sodium sulfate, OTC: over-the-counter, PEG: polyethylene glycol

Of the 1005 patients who reported a prior history of bowel preparation use, a total of 851 (84.7%) stated that their experience with NER1006 was much better/better (*n* = 656; 65.3%) or about the same (*n* = 195; 19.4%) compared with bowel preparations they had previously used (Table 2). The majority of patients reported that their experience with NER1006 was much better/better than their previous experience with 4 L PEG (68.6% [120/175]), oral sulfate solution (61.6% [106/172]), an over-the-counter bowel preparation (e.g., PEG 3350; 52.7% [68/129]), or 2 L PEG (71.3% [82/115]; Fig. 3). These four bowel preparations were the most common types that had been used previously.

Trends in survey responses were similar in subgroup analyses by sex (male vs female), colonoscopy indication (diagnostic vs routine screening), and age (< 65 years vs ≥ 65 years), with some larger differences observed in the male versus female subgroups (Table 3). For example, a higher percentage of men compared with women completed all the bowel preparation (94.6% vs 83.1%), found it very easy to prepare and take NER1006 (81.4% vs 73.5%), and were very satisfied with NER1006 overall (69.7% vs 59.3%).

**Table 3:** Patient survey results, subgrouped by sex, colonoscopy indication, or age group

Topic and response categories	Sex, n (%)		Colonoscopy indication, n (%)		Age, n (%)	
	*n* = 1556^a^		*n* = 1598		n = 1598	
	Males	Females	Diagnostic	Routine Screening	< 65 y	≥ 65 y
	*n* = 558	*n* = 998	*n* = 557	*n* = 1041	*n* = 1189	*n* = 409
Volume of bowel preparation regimen completed						
All	528 (94.6)	829 (83.1)	484 (86.9)	908 (87.2)	1022 (86.0)	370 (90.5)
Most	27 (4.8)	136 (13.6)	58 (10.4)	109 (10.5)	135 (11.4)	32 (7.8)
At least half	2 (0.4)	25 (2.5)	11 (2.0)	18 (1.7)	22 (1.9)	7 (1.7)
Less than half	1 (0.2)	8 (0.8)	4 (0.7)	6 (0.6)	10 (0.8)	0
“How easy was it for you to prepare and take [NER1006]?”						
Very easy (score, 7–9)	454 (81.4)	734 (73.5)	414 (74.3)	802 (77.0)	906 (76.2)	310 (75.8)
Medium level of difficulty (score, 4–6)	73 (13.1)	164 (16.4)	85 (15.3)	160 (15.4)	184 (15.5)	61 (14.9)
Very difficult (score, 1–3)	31 (5.6)	100 (10.0)	58 (10.4)	79 (7.6)	99 (8.3)	38 (9.3)
How important is it to you that using [NER1006] required only 64 oz of total solutions?”						
Very important (score, 7–9)	405 (72.6)	774 (77.6)	413 (74.1)	793 (76.2)	884 (74.3)	322 (78.7)
Medium importance (score, 4–6)	114 (20.4)	159 (15.9)	102 (18.3)	181 (17.4)	220 (18.5)	63 (15.4)
Not important (score, 1–3)	39 (7.0)	65 (6.5)	42 (7.5)	67 (6.4)	85 (7.1)	24 (5.9)
“How important is it to you that [NER1006] can be taken with your choice of clear liquids?”						
Very important (score, 7–9)	402 (72.0)	762 (76.4)	418 (75.0)	774 (74.4)	888 (74.7)	304 (74.3)
Medium importance (score, 4–6)	104 (18.6)	159 (15.9)	92 (16.5)	181 (17.4)	208 (17.5)	65 (15.9)
Not important (score, 1–3)	52 (9.3)	77 (7.7)	47 (8.4)	86 (8.3)	93 (7.8)	40 (9.8)
“How satisfied were you with the taste of [NER1006]?”						
Very satisfied (score, 7–9)	215 (38.5)	327 (32.8)	192 (34.5)	363 (34.9)	412 (34.7)	143 (35.0)
Moderately satisfied (score, 4–6)	199 (35.7)	335 (33.6)	180 (32.3)	370 (35.5)	408 (34.3)	142 (34.7)
Not satisfied (score, 1–3)	144 (25.8)	336 (33.7)	185 (33.2)	308 (29.6)	369 (31.0)	124 (30.3)
“How satisfied were you with [NER1006] overall?”						
Very satisfied (score, 7–9)	389 (69.7)	592 (59.3)	327 (58.7)	677 (65.0)	752 (63.2)	252 (61.6)
Moderately satisfied (score, 4–6)	130 (23.3)	288 (28.9)	174 (31.2)	259 (24.9)	323 (27.2)	110 (26.9)
Not satisfied (score, 1–3)	39 (7.0)	118 (11.8)	56 (10.1)	105 (10.1)	114 (9.6)	47 (11.5)
How satisfied were you with your health care provider for having prescribed [NER1006]?”						
Very satisfied (score, 7–9)	452 (81.0)	744 (74.5)	410 (73.6)	814 (78.2)	909 (76.5)	315 (77.0)
Moderately satisfied (score, 4–6)	83 (14.9)	202 (20.2)	117 (21.0)	179 (17.2)	225 (18.9)	71 (17.4)
Not satisfied (score, 1–3)	23 (4.1)	52 (5.2)	30 (5.4)	48 (4.6)	55 (4.6)	23 (5.6)
“Would you be willing to recommend [NER1006] to family/friends?”						
Yes	391 (70.1)	616 (61.7)	340 (61.0)	687 (66.0)	770 (64.8)	257 (62.8)
Maybe	132 (23.7)	262 (26.3)	165 (29.6)	249 (23.9)	316 (26.6)	98 (24.0)
No	35 (6.3)	120 (12.0)	52 (9.3)	105 (10.1)	103 (8.7)	54 (13.2)
“How was your experience with [NER1006] compared to the other bowel cleansing medications(s) you previously used?^a^						
	*n* = 329	*n* = 654	*n* = 301	*n* = 704	*n* = 629	*n* = 376
Much better/better	237 (72.0)	408 (62.4)	189 (62.8)	467 (66.3)	402 (63.9)	254 (67.6)
About the same	53 (16.1)	134 (20.5)	59 (19.6)	136 (19.3)	126 (20.0)	69 (18.4)
Worse/much worse	39 (11.9)	112 (17.1)	53 (17.6)	101 (14.3)	101 (16.1)	53 (14.1)

^a^Sex not reported (i.e., unknown) for 42 patients

## Discussion

Adequate bowel preparation is an important metric for achieving high-quality colonoscopy and enhancing detection of precancerous lesions and CRC [15, 16]. Colonoscopy success is largely dependent on patient factors, particularly with respect to their acceptance of and adherence to the bowel preparation regimen [15, 17]. Because bowel preparations vary in volume, palatability, and taste, it is important to continue to gather insight into patient attitudes and preferences related to bowel preparations for colonoscopy. Therefore, this online survey was conducted to assess patient tolerability and acceptability of a US Food & Drug Administration–approved 1 L PEG formulation, NER1006, across a wide US geographic population undergoing colonoscopy.

The survey indicated that most patients reported NER1006 as very easy to prepare and take, and most patients were very or moderately satisfied with NER1006. Results were generally similar when assessed by sex, colonoscopy indication (diagnostic vs routine screening), or age (< 65 years vs ≥ 65 years), although some larger percentage differences were observed between males and females. The results supported clinical trial data that indicated NER1006 patient satisfaction was favorable and adherence was high. Phase 3 clinical trial data from the DAYB (day before arm) and MORA (morning arm) trials indicated that 97.0% to 99.1% of NER1006–treated patients said it “was not very difficult to follow the instructions,” 94.5% to 95.9% of patients indicated that NER1006 taste was “not very unacceptable,” and 92.2% to 94.5% of patients noted that NER1006 bowel preparation regimen was “not very difficult to drink” [12, 13]. In the phase 3 NOCT (nocturnal pause arm) trial, a similar percentage of patients receiving NER1006 compared with those receiving oral sodium sulfate reported that NER1006 was “very easy” or “quite easy” to drink (56.2% vs 52.1%, respectively; *P* = 0.35), and a significantly higher percentage of NER1006–treated patients indicated the taste was “very acceptable” or “acceptable” compared with ratings of oral sodium sulfate–treated patients (66.7% vs 50.7%; *P* = 0.0001) [11].

In a study of 5000 patients undergoing screening colonoscopy, ~ 20% of patients stated that bowel preparation was one of the most worrisome factors before the planned procedure [18]. The authors of that study concluded that optimizing the taste of bowel preparations and the required volume intake would likely increase participation rates for screening colonoscopy [18]. The current survey found that that most (84.7%) patients who reported previously taking a bowel preparation believed their experience with NER1006 was much better/better or about the same compared with that for the previously administered product.

Although efficacy conclusions cannot be drawn from data obtained via the current survey, it is reassuring that most patients considered their experience with NER1006 to be better or at least similar to previous experiences with other bowel preparations. Whether a positive NER1006 experience might translate to increased adherence with future screening or surveillance recommendations by health care providers remains unknown and warrants further study.

Limitations of the current survey include parameters intrinsic to any survey-based study design, such as potential for sampling bias and/or recall bias [19, 20]. For example, patients may be more likely to participate in and complete an online survey because they are interested in the topic or are attracted by the incentive offered for survey completion. In addition, lack of a comparator group, information on total amount of NER1006 or additional clear fluids consumed, measurement of safety outcomes, and completion of health care provider assessments (e.g., quality of bowel preparation, neoplasm detection rate) are other limitations.

## Conclusion

In this first real-world, multicenter US survey, patient experience with NER1006 as a bowel preparation for colonoscopy was favorable and adherence was high. The majority of patients were very or moderately satisfied with the overall experience. Studies evaluating real-world evidence for NER1006, including efficacy, such as adenoma and polyp detection rates during colonoscopy, are warranted.

## Supplementary Information

**Additional file 1. Appendix**: Online survey questions completed by participants.

## Data Availability

All data generated or analyzed during this survey and used in the study are available from the corresponding author upon reasonable request.

## Abbreviations

CI: Confidence interval
CRC: Colorectal cancer
DAYB: Day before arm
MORA: Morning arm
NOCT: Nocturnal pause arm
OR: Odds ratio
OSS: Oral sodium sulfate
OTC: Over-the-counter
PEG: Polyethylene glycol
