# Supplementary material for: Patient experience with NER1006 as a bowel preparation for colonoscopy: a prospective, multicenter US survey
Source: BMC Gastroenterol. 2021 Feb 15;21:70. doi: 10.1186/s12876-021-01605-y (PMC7885614; doi:10.1186/s12876-021-01605-y)
Supplement: Supplementary file 1 — Additional file 1. Appendix: Online survey questions completed by participants. [file 12876_2021_1605_MOESM1_ESM.docx]

**Appendix: Online Survey Questions**

- Do you have a family history of colon cancer?
  (Yes/No)
- What is the reason for your colonoscopy procedure?
  (Routine screening/Diagnostic screening)
- Is this your first colonoscopy?
  (Yes/No)
- Which one of these bowel cleansing medications did you use for a prior colonoscopy? Select all that apply. You must select at least one.
  (Suprep^®^/MoviPrep^®^/Prepopik^®^/Clenpiq^®^/GoLYTELY^®^/Another 4-liter bowel cleansing solution/OTC medication [such as MiraLAX^®^]/Another prescription medication/I don't remember)
- How did you take Plenvu?
  (2-day split dose/Same-day morning of the colonoscopy)
- Did you use the instruction sheet that came with the Plenvu packaging to help you complete the dose?
  (Yes/No)
- How much of the regimen did you complete?
  (Entire/Most/At least half/Less than half)
- How easy was it for you to prepare and take Plenvu?
  (Choose any number from 1 to 9, with 1 being very difficult and 9 being very easy.)
- How important is it to you that using Plenvu required only 64 ounces of total solutions?
  (Choose any number from 1 to 9, with 1 being not important at all and 9 being very important.)
- How important is it to you that Plenvu can be taken with your choice of clear liquids?
  (Choose any number from 1 to 9, with 1 being not important at all and 9 being very important.)
- How satisfied were you with the taste of Plenvu?
  (Choose any number from 1 to 9, with 1 being not satisfied at all and 9 being very satisfied.)
- How satisfied were you with Plenvu, overall?
  (Choose any number from 1 to 9, with 1 being not satisfied at all and 9 being very satisfied.)
- How satisfied are you with your healthcare provider for having prescribed Plenvu?
  (Choose any number from 1 to 9, with 1 being not at all satisfied and 9 being very satisfied.)
- How has your experience with Plenvu compared to the other bowel cleansing medication(s) you previously used?
  (Much better/Better/About the same/Worse/Much worse)
- Would you be willing to recommend Plenvu to family/friends?
  (Yes/Maybe/No)
- Would you recommend others planning to have a colonoscopy speak with their healthcare provider about Plenvu?
  (Yes/Maybe/No)
- Do you think participating in this program will help you better communicate with your healthcare provider about your condition and your treatment experience?
  (Yes/Maybe/No)
- What motivated you most to participate in this program?
  (To provide more information to my healthcare provider/Because my healthcare provider
  asked me to/The $20 Amazon.com gift card/Another reason)
- Would you participate in a program like this again?
  (Yes/Maybe/No)
